# The Red Color of Life Transformed – Synthetic Advances and Emerging Applications of Protoporphyrin IX in Chemical Biology

**DOI:** 10.1002/ejoc.202000074

**Published:** 2020-03-30

**Authors:** Elisabeth Sitte, Mathias O. Senge

**Affiliations:** ^1^ School of Chemistry Trinity College Dublin The University of Dublin Trinity Biomedical Sciences Institute 152‐160 Pearse Street 2 Dublin Ireland; ^2^ Institute for Advanced Study (TUM‐IAS) Technische Universität München Lichtenberg‐Str. 2a 85748 Garching Germany

**Keywords:** Catalysis, Chemical Biology, Heme, Porphyrinoids, Protoporphyrin IX, Sensing

## Abstract

Protoporphyrin IX (PPIX) is the porphyrin scaffold of heme b, a ubiquitous prosthetic group of proteins responsible for oxygen binding (hemoglobin, myoglobin), electron transfer (cytochrome c) and catalysis (cytochrome P450, catalases, peroxidases). PPIX and its metallated derivatives frequently find application as therapeutic agents, imaging tools, catalysts, sensors and in light harvesting. The vast toolkit of accessible porphyrin functionalization reactions enables easy synthetic modification of PPIX to meet the requirements for its multiple uses. In the past few years, particular interest has arisen in exploiting the interaction of PPIX and its synthetic derivatives with biomolecules such as DNA and heme‐binding proteins to evolve molecular devices with new functions as well as to uncover potential therapeutic toeholds. This review strives to shine a light on the most recent developments in the synthetic chemistry of PPIX and its uses in selected fields of chemical biology.

## Graphical abstract

The use of the heme precursor protoporphyrin IX for biological applications has experienced a steep increase in recent years. Herein, we review possibilities for synthetic modification of this porphyrin scaffold and discuss its emerging key uses in the field of chemical biology.

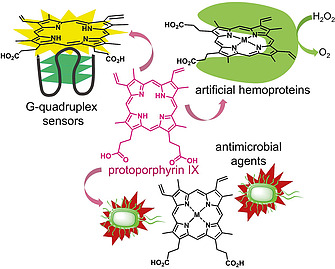

## 1. Introduction

Blood is red and grass is green. This epitome highlights the two main fundamental classes of porphyrinoids all around us, the red porphyrins and green chlorophylls as the pigments of life.^1^ A key compound is protoporphyrin IX (PPIX), which, as the biosynthetic precursor of hemes as well as the chlorophylls of photosynthesis, remains one of the most intensely studied natural tetrapyrrolic compounds. In daily life, next to blood, it is often found on the breakfast table as the shell pigment in brown eggs.^2^

PPIX and its derivatives have been used in many disciplines such as medicine, catalysis, sensing, optical materials, and chemical biology. The development of new applications for PPIX, notably in recent years, is accompanied by advancements of functionalization reactions as well as new methods developments for the total synthesis of this porphyrin.

Tetrapyrrole chemistry is a field that is highly inspired by biological processes. These complex molecules were naturally evolved to serve unique functions in living organisms. The corrinoid vitamin B_12_ is a precursor of coenzyme B_12_ and methylcobalamin, two enzyme cofactors,^3^ factor F430 functions in methanogenic bacteria,^4^ while chlorophylls and bacteriochlorophylls act as the photosynthetic pigments in plants and bacteria.^1^ Hemes are iron‐containing tetrapyrroles that are prosthetic groups of proteins and enzymes that exert a variety of functions. Prominent examples are hemoglobin and myoglobin that transport oxygen by its binding to the Fe center of heme, cytochromes of type c which are involved in the electron transport chain, cytochromes of the P450 family that oxygenize organic substrates and are involved in biosynthesis as well as drug metabolism, and catalases and peroxidases which oxidize substrates using H_2_O_2_.^5^

**Figure 1:**
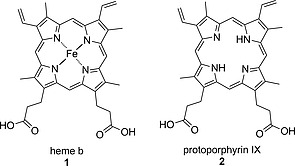
Heme b (**1**) and protoporphyrin IX (**2**).

Heme b (**1**) is the most abundant of the hemes. It consists of a porphyrin ligand, protoporphyrin IX (**2**), and a Fe(II) center (Figure 1). The porphyrin macrocycle carries two propionate, two vinyl, and four methyl groups in the β‐positions while the meso‐positions remain free. While the heme metal center imparts anchoring to proteins by axial ligation to amino acid residues, resulting in penta‐ or hexacoordinated Fe,[5a] the peripheral substituents on the porphyrin core can also play a role in fixing the prosthetic group inside the heme‐binding pocket of the protein. The propionate moieties of heme have been shown to engage in electrostatic interactions such as hydrogen bonds with different residues of the protein backbone,^6^ whereas the vinyl groups can form covalent bonds with cysteine residues, generating so‐called heme c.[5a]

The challenging synthesis of the pigments of life has stimulated chemists' ambitions since the early days of the 20^th^ century. Significant contributions in the field were made by Fischer, who developed total syntheses of PPIX,^7^ and later by MacDonald,^8^ Lindsey,^9^ and others.^10^ Additionally, facile functionalization of the porphyrin macrocycle is possible, mostly via organometallic reactions.^11^

However, the multi‐step total synthesis of natural porphyrins such as PPIX remains tedious, owing to the three different types of substituents in the β‐positions and the unsymmetrical substitution pattern.^10^ It involves the synthesis of different pyrrole and dipyrromethane building blocks which requires specific substituents in the α‐positions to ensure correct assembly of the porphyrin core. The sensitivity of the vinyl groups and the inconvenience of handling the propionate moieties necessitates for these groups to be installed at the end of the synthesis.^12^ Nevertheless, the natural abundance of heme in hemoglobin enables the isolation of PPIX from blood in significant amounts,^13^ which results in the commercial availability of PPIX and several PPIX metal complexes. For this reason, synthetic efforts have mostly been focused on the modification of the periphery of PPIX.^14^

Today PPIX is of considerable interest for different scientific communities, be it biologists, chemists, physicists or materials scientists and the past three decades have seen a steep increase in related publications. PPIX combines different powerful assets that facilitate its use in different areas. As an important natural product, it provides biocompatibility and can interact with biomolecules; thus, it can be used to modulate molecular or cellular functions.^15^ Currently, it is widely studied in biomedicine for applications such as photodynamic therapy (PDT),^16^ antimicrobial and antiviral phototherapy,^17^ biosensing,^18^ bioimaging,^19^ catalysis^20^ and interference with biochemical pathways.^21^ Additionally, the inherent properties of PPIX as a porphyrin, the wide range of biological functions manifested in nature through manipulation of the macrocycle conformation,^22^ its ready availability and the possibility of facile attachment of groups at its propionate moieties^14^ renders it a compound frequently employed for a variety of other applications such as sensing,^23^ light harvesting,^24^ electrochemistry^25^ as well as uses in supramolecular chemistry^26^ which have been reviewed here by Bhosale et al.^27^

The range of established uses and the emergence of many new applications notably at the interface of chemistry and biology prompted us to review the relevant literature. Here, we aim to find a middle ground between updating the specialist reader on recent advances in the synthetic chemistry of PPIX and, simultaneously, to discuss some of its key uses in modern chemical biology applications.

## 2. Synthesis of PPIX Derivatives

### 2.1. Total Synthesis

**Scheme 1:**
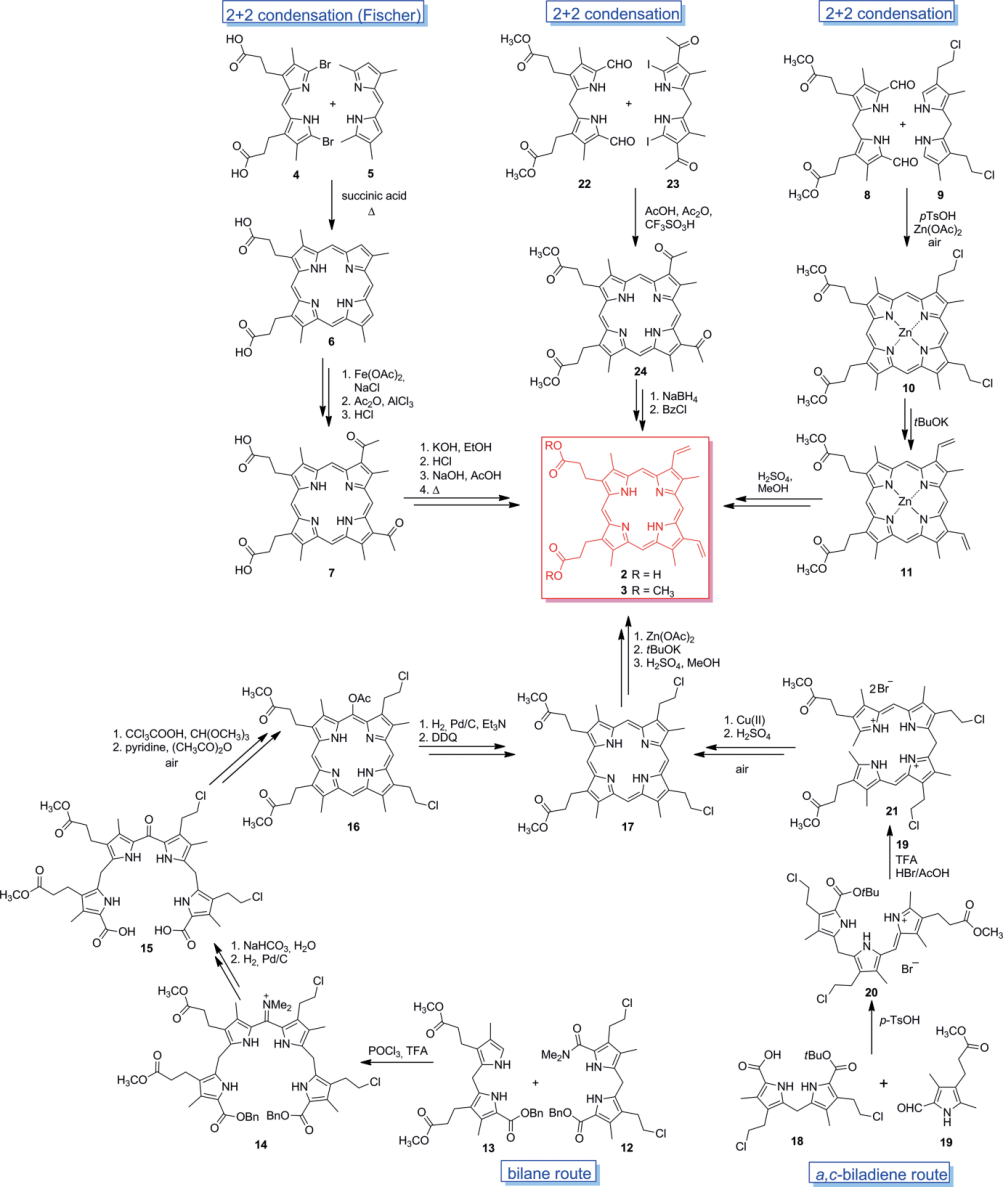
Different routes towards the total synthesis of PPIX (**2**) and PPIX dimethyl ester (**3**).

In the 1920s the Munich‐based chemist Hans Fischer extensively investigated the total synthesis of tetrapyrroles. Highlights were the synthesis of deuteroporphyrin IX in 1928,^31^ followed by the first syntheses of PPIX, hematoporphyrin and hemin one year later.^7^ The synthesis of the unsymmetrically β‐substituted PPIX, which earned the German chemist the Nobel Prize for Chemistry in 1930, was intricate and lengthy. It started with the synthesis of different pyrroles that were reacted to yield the 5,5'‐bromo‐ and 5,5'‐methyl‐substituted dipyrromethenes **4** and **5**, respectively, which were condensed in a succinic acid melt to give deuteroporphyrin IX (**6**) (Scheme 1). Iron(III) insertion, followed by acetylation and reduction yielded hematoporphyrin (**7**), which was converted to PPIX (**2**) by dehydration.^7^, ^28^

Later, approaches for the de novo synthesis of PPIX were developed, mainly by Smith and co‐workers.[12a], [12b], 29, 30 To prepare deuterated derivatives of PPIX dimethyl ester (PPIX‐dme, **3**), Smith applied different strategies. One comprised a MacDonald‐type^8^ 2+2 condensation of 5,5'‐formyl‐dipyrromethane **8** with 5,5'‐unsubstituted dipyrromethane **9**. The cyclization to **10** was achieved by *p*‐toluenesulfonic acid and zinc acetate. Hereafter, the 3,8‐vinyl groups were generated by base‐catalyzed dehydrochlorination at the 2‐chloroethyl moieties of **10**, followed by demetallation and esterification.[12a] Alternatively, **2** was prepared via condensation of the phosphoryl chloride complex of amido‐dipyrromethane **12** with 5‐unsubstituted dipyrromethane** 13** to give bilane **14** which was further converted into the biscarboxylate **15** and cyclized using trichloroacetic acid and trimethyl orthoformate. Acetylation to give **16**, hydrogenation and reoxidation of the porphyrin macrocycle yielded **17** as a precursor for PPIX‐dme.[12a]

A different approach for the synthesis of **2** involved the formation of tripyrrene **20** and then *a,c*‐biladiene **21** from dipyrromethane **18** and subsequent condensations with formyl‐pyrrole **19**. Cyclization of the linear tetrapyrrole using a copper(II) salt and demetallation afforded **17**, which can be converted to **1**.[12b], [30a], [30b], [30c], [30e], [30f], [30g], [30h], [30i], [30j], 31 More recently, the PPIX dimethyl ester precursor **24** was prepared from a condensation of 5,5'‐diiodo‐ and 5,5'‐diformyl‐dipyrromethanes **22** and **23** by Martin et al.[12c] The vinyl groups were obtained by reduction and dehydration of acetyl moieties in the 3,8‐positions of the porphyrin ring.

### 2.2. Substituent Modifications

#### 2.2.1 Classic Functionalization Reactions

**Scheme 2:**
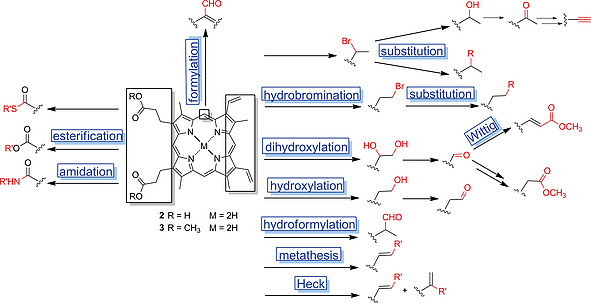
Selected substituent and macrocycle modification on PPIX (**2**), PPIX‐dme (**3**) and their metal complexes.

The interest in PPIX has led to the development of a range of functionalization reactions for the vinyl groups and propionic acid moieties, the majority of whom has been discussed in a comprehensive review by Pavlov.^14^ A selection of frequently applied functionalization reactions for PPIX (**2**), PPIX‐dme (**3**) and their metal complexes, is given in Scheme 2. Among the vinyl group derivatizations are hydrobromination using hydrobromic acid in combination with acetic acid and subsequent nucleophilic substitution,^14^, ^32^ mono‐ and dihydroxylation^14^, [32b], [32c], 33 and subsequent conversion to aldehydes,^14^, [32b], 33 acetyls,^14^, ^34^ alkynes,^14^, ^35^ esters^14^, [32b], 36 as well as hydroformylation.^37^

Metal‐catalyzed C–C bond formation reactions have been performed on PPIX vinyl groups, including olefin metathesis^17^, ^41^ and Heck reactions.^14^, ^39^ Multiple PPIX conjugates have been prepared by ester,^10^, [33b], 40 thioester^14^, ^41^ and amide^10^, [32e], [40b], 42 formation at the carboxylic moieties (Scheme 2). The free meso‐positions of PPIX can be formylated using a Vilsmeier reaction whereupon regioisomeric mixtures are obtained.^43^ Although long‐established reactions such as amide couplings form the basis for most of PPIX synthetic chemistry and are widely applied for the generation of functional derivatives, some advances to broaden the synthetic spectrum of **2** and **3** have been made more recently and are detailed in the following sections.

#### 2.2.2 Modifications of the Vinyl Groups

A new method for the conversion of the vinyl groups of **3** to formyl and acetyl moieties, respectively, while circumventing the use of OsO_4_ was introduced by Oba and co‐workers.^44^ The reaction of the vinyl groups with iodine and PIFA in ethylene glycol/1,2‐dichloroethane gave a diiodoether porphyrin which was converted to 3,8‐diacetyl porphyrin. An iodohydrin porphyrin was also synthesized in a similar manner which was converted into the 3,8‐diformyl derivative of PPIX‐dme.

**Scheme 3:**
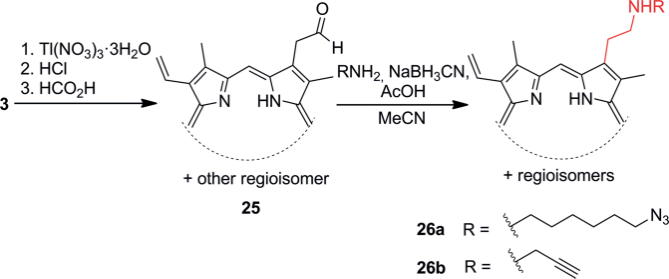
Synthesis of amine‐substituted PPIX derivatives via condensation with monoformyl‐PPIX dimethyl ester.

PPIX dimethyl ester monoaldehyde **25**, synthesized as previously reported,^32^, ^45^ was condensed with amines yielding azido‐ and alkynyl‐functionalized porphyrin derivatives, e.g., **26a** and **26b** (Scheme 3). Subsequent copper‐catalyzed azide‐alkyne cycloaddition with vitamin B_12_ derivatives afforded molecular hybrid molecules.^46^

**Scheme 4:**
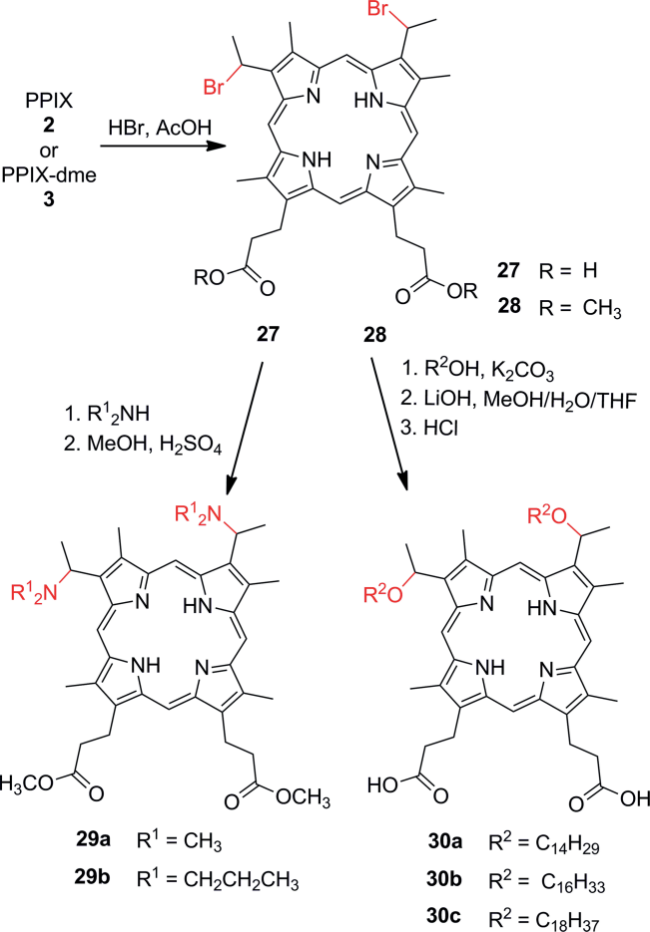
Nucleophilic substitution reactions on dibromo‐porphyrins **27** and **28** with amines and alcohols.

As mentioned previously, hydrobromination of the vinyl groups, followed by nucleophilic substitution is a versatile tool for addition of various substituents to the vinyl‐half of PPIX.^32^ This reaction was used to prepare tertiary alkylamine‐substituted PPIX dimethyl esters **29a** and **29b** from **27** (Scheme 4). These compounds were used in a study on the influence of the amine substituents on the hydrophobicity and phototoxicity of photosensitizers.^47^ Dibromo porphyrin **28** was also reacted with long aliphatic alcohols to obtain PPIX lipids **30a**–**30c**. These photosensitizer‐appended lipids were assembled into liposomes, forming platforms for sensitizer delivery in aqueous solution.^48^ Bishydroxylation of the vinyl groups of PPIX‐dme gave the tetraalcohol[33a] as a useful building block which was further reacted with carborane carbonyl chloride to obtain a porphyrin carrying four carboranyl esters.[34a], 49 Boronated porphyrins are used in boron neutron capture therapy and PDT.

**Scheme 5:**
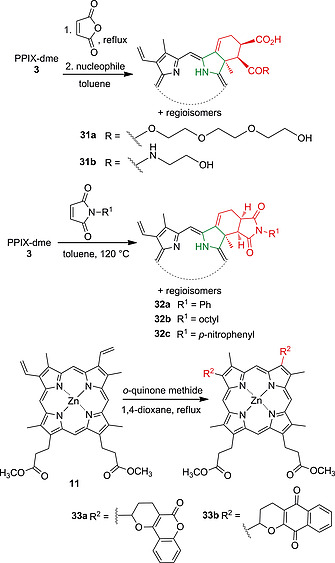
Cycloaddition reactions of the vinyl groups of PPIX‐dme **3** and its Zn(II) complex **11**.

Electrocyclic reactions involving the vinyl groups of PPIX‐dme with diazo compounds are known to produce chlorins.^14^, ^50^ This was expanded by Cavaleiro's group who reacted **3** with maleic anhydride, followed by the addition of nucleophiles to the anhydride moiety giving amphiphilic chlorins e.g. **31a** and **31b**.^51^ Similarly, Uchoa et al. synthesized photodynamically active chlorins by a Diels‐Alder reaction of **3** with maleimides. The cycloaddition products **32a**–**32c** did not show any π‐π‐stacking induced self‐aggregation due to their *endo* conformation.^52^ A combined Knoevenagel hetero Diels‐Alder reaction on the vinyl groups of Zn complex **11** [Zn(II)PPIX‐dme] with coumarin, quinolone, and naphthoquinoline derivatives was carried out in order to obtain new photosensitizer‐natural product conjugates, e.g. **33a** and** 33b** (Scheme 5).^53^

The PPIX vinyl groups are also suitable sites for olefin cross‐metathesis reactions and have been subjected to the attachment of different aliphatic side chains using a second‐generation Grubbs catalyst.^38^ Cavaleiro and co‐workers synthesized glycoprotoporphyrin derivatives as photosensitizers with increased water‐solubility and membrane interaction by this method.^54^ Tetraalkyl‐substituted, as well as amphiphilic PPIX derivatives, were synthesized similarly. The introduced tails mediated solvophobicity‐induced self‐assembly of the porphyrins.^55^

The reduction of the vinyl to ethyl moieties yields mesoporphyrin IX (MPIX). This derivative with better stability than PPIX has traditionally been prepared using Pd‐ and Pt‐catalysts.[33b], 56 More recently, reduction was achieved by RuCl_3_‐catalyzed hydrogenation with H_2_ in *N*,*N*‐dimethylacetamide.^57^ Several metal complexes of **3** were found to self‐catalyze hydrogenation of their vinyl groups in a CoCl_2_/NaBH_4_ system.^58^

The tendency of vinyl groups to undergo radical additions was exploited to synthesize PPIX containing polymers by free‐radical copolymerization.^59^ The reaction of **2** with monomers such as acrylamide, methacrylic acid, and *N*,*N'*‐methylenebisacrylamide is achieved using radical initiators and the resulting polymers were used for spectrophotometric detection of metal ions[59a], [59b] and as a hydrogel‐based pH sensor in a glass electrode.[59c] Incorporation of **2** or **3** in poly(*N*‐isopropylacrylamide) hydrogels circumvented the low water‐solubility of these photosensitizers and their tendency to form aggregates in polar solvents.[59d], [59e] Such biocompatible hydrogels show potential for the clinical administration of PPIX as a photosensitizer.

**Scheme 6:**
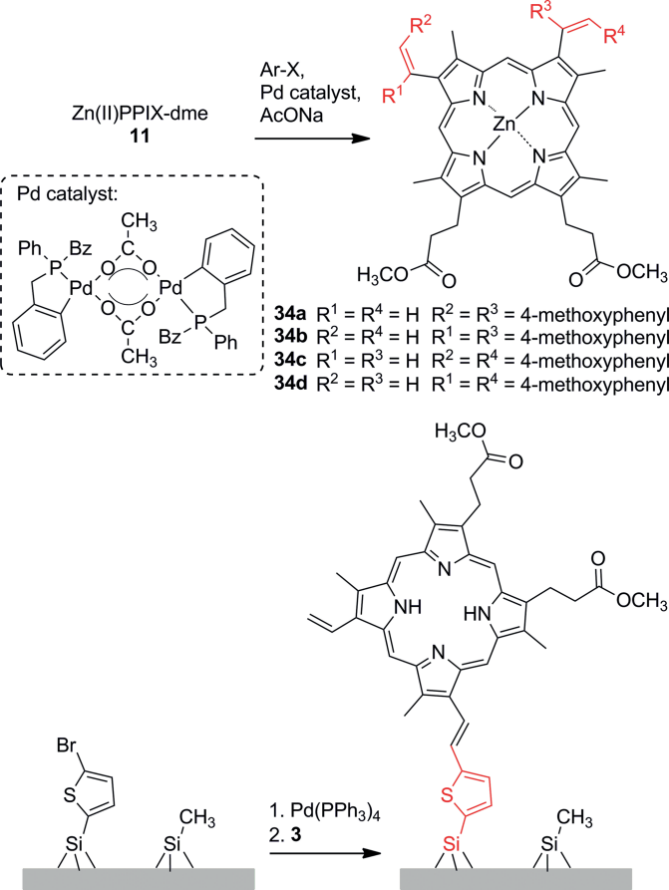
Heck reactions using PPIX dimethyl ester and Zn(II)PPIX‐dme.

Heck coupling reactions on the vinyl groups of **3** were first carried out by using mercurated arenes as coupling substrates.[39a], [39c] Later, Castella et al. implemented a Pd‐catalyzed coupling reaction of **11** and different halo‐aryl compounds which yielded mixtures of regioisomers **34a**–**34d**.[39b] A similar approach was taken to immobilize PPIX‐dme on silicon surfaces. This involved the bromination of thienyl‐functionalized surfaces using NBS and subsequent coupling of the bromo‐heteroarenes with **3** using Pd(PPh_3_)_4_ (Scheme 6).^60^

**Scheme 7:**
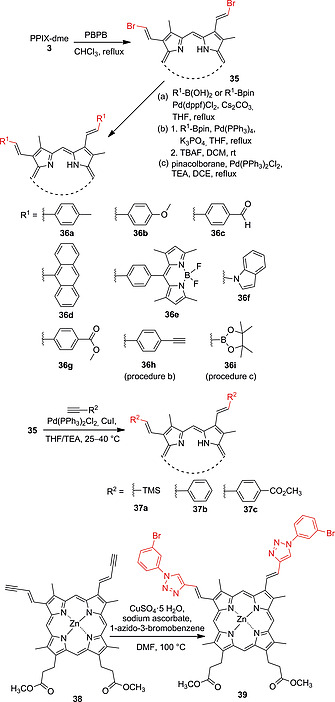
Bromination of PPIX dimethyl ester vinyl groups and Suzuki‐Miyaura, Sonogashira and 1,3‐dipolar cycloaddition reactions for vinyl group extension.

Our group recently looked into the use of different Pd‐catalyzed coupling reactions for the modification of the vinyl groups of PPIX dimethyl ester to achieve better regioselectivity.^61^ Bromination of **3** using pyridinium bromide perbromide (PBPB) led to regio‐ and stereoselective formation of dibromo derivative **35** (Scheme 7). This precursor was used for Suzuki‐Miyaura and Sonogashira coupling reactions^62^ to produce a library of PPIX derivatives appended with aromatic **36a**–**36i** or alkynyl moieties **37a**–**37c**. The ethynyl‐substituted Zn(II) porphyrin **38** was further reacted with 1‐azido‐3‐bromobenzene in a Cu‐catalyzed 1,3‐dipolar cycloaddition to afford arm‐extended porphyrin **39**. These methods allow for easy and versatile modifications of PPIX‐dme.^61^

PPIX vinyl groups can also be reacted with thiols to give Markovnikov addition products.^63^ In the same manner, iron(III) PPIX (hemin) was immobilized on gold surfaces functionalized with 1,6‐hexanedithiol. The assembly was used as an iron(III)/iron(II) PPIX redox electrode.^64^

#### 2.2.3 Modifications of the Propionic Acid Moieties

Modification of the propionic acid moieties presents the simplest means to transform **2** and is frequently applied for the generation of porphyrin derivatives with specific functionalities and for the attachment of porphyrins to molecules and materials. Reactions at the carbonyl group such as esterifications and amidations are usually straightforward and can be used for the synthesis of a wide range of porphyrin derivatives. Hence, peptide coupling reactions with PPIX are well established in the literature.^14^, [32e], [40b], 42 This type of functionalization has remained the main route for the synthesis of PPIX derivatives, be it for the generation of therapeutics,[16e], 41, 42, 47, 65 imaging,[65d], 66 sensing,^67^ materials chemistry,^68^ photooxidation catalysis,^69^ use in electrochemistry,^70^ as biomimetics,^71^ as modulated biomolecules^72^ and for supramolecular assemblies.^55^, ^73^

**Scheme 8:**
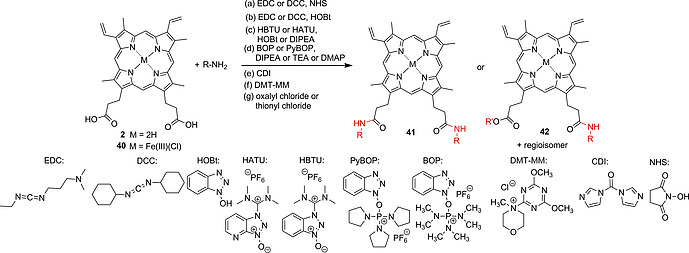
Different strategies for amide bond formation at the PPIX carboxylic acid moieties.

Various strategies were applied to effect amide bond formation on **2** or hemin (**40**) (Scheme 8). Activation of the carboxylic acid by reaction with carbodiimides such as 1‐ethyl‐3‐(3‐dimethylaminopropyl)carbodiimide (EDC)[16e], [65b], [65c], [65f], [65j], [65t], [65u], [65v], 74 or *N*,*N'*‐dicyclohexylcarbodiimide (DCC),[65d], [65l], 66, [73a] respectively, and subsequent formation of the *N*‐hydroxysuccinimide (NHS) esters, followed by reaction with the amine coupling substrates, reliably yielded PPIX mono‐ and bisamide products **41** and **42**.

Alternatively, DCC[42a], [65d], [65r], 67 and EDC^55^, [73b], [73d] were used in combination with HOBt to form active carboxylate esters which easily reacted with amines. In other cases, amide bond formation on PPIX and iron(III) PPIX was effected by use of the potent peptide coupling agents hexafluorophosphate azabenzotriazole tetramethyl uronium (HATU)[65k], [65n] and hexafluorophosphate benzotriazole tetramethyl uronium (HBTU),[65i], [65p], [69b], 71, 75 respectively, in combination with a tertiary amine base. Dorota Gryko and co‐workers screened different conditions for the coupling of amino acids to PPIX. The best yields were obtained using a combination of HBTU, HOBt, and *N*,*N*‐diisopropylethylamine (DIPEA).[75]

**Scheme 9:**
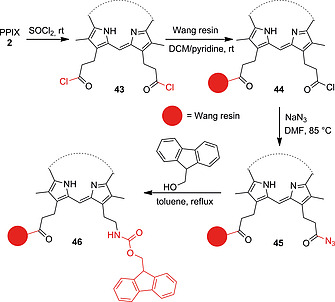
Synthesis of PPIX monoacyl azide **45** on solid support and Curtius rearrangement to give inverted amide **46**.

HBTU/DIPEA,[65a], [65h] HBTU/HOBt[65i], [65p] and HATU/DIPEA[65n] also proved as efficient activating agents in solid‐phase‐based peptide couplings with PPIX. Fmoc‐protected peptides immobilized on rink amide,[65a], [65i] Wang‐type[65n] or 2‐chlorotrityl chloride[65h], [65p] resins were deprotected, followed by reaction of the free amino groups with the activated PPIX esters. Cleavage from the resin gave porphyrins with targeting peptide moieties suitable for cancer PDT and antimicrobial PDT. A different strategy is the conversion of carboxylic acids to acyl chlorides via the use of oxalyl chloride^41^, [65e] or thionyl chloride.^47^, [65w], [68a], [73c] Sol and co‐workers treated PPIX with thionyl chloride to obtain the diacyl chloride porphyrin derivative **43** (Scheme 9). One of the activated carboxyl moieties was then used for immobilization of PPIX on a Wang‐type resin (**44**) whereas the free acyl chloride group was converted into an acyl azide moiety (**45**) followed by a Curtius rearrangement to its corresponding isocyanate derivative. This was converted to fluorenylmethyloxycarbonyl (Fmoc)‐protected porphyrin monoamide on solid support (**46**).[65w]

The reaction of amines with PPIX was also effected using benzotriazol‐1‐yloxytris(dimethylamino)phosphonium hexafluorophosphate (BOP)[68b] and the related (benzotriazol‐1‐yl‐oxytripyrrolidinophosphonium hexafluorophosphate) (PyBOP)[65m], [65w], [72b] in combination with DIPEA, triethylamine (TEA) or DMAP as base catalysts. Protection of one of the PPIX carboxylic acid moieties by esterification with *tert*‐butanol before carrying out the amide coupling can allow for the synthesis of monoamides.[70b], [72b], [73e]

Activation of the carboxylic acid moieties with carbonyldiimidazole (CDI) proved to be an efficient method to immobilize PPIX and hemin on amine‐functionalized surfaces of cellulose[65q], [68c] and a poly(ethylene‐*co*‐methacrylic acid) membrane.[69a] The PPIX‐functionalized materials show potential for antibacterial PDT[65q] and as photooxidation catalysts.[69a]

Alternative strategies for PPIX peptide couplings involve DMT‐MM‐mediated activation of the carboxylic acid moieties,[65g] conversion of the acids to mixed carboxylic anhydrides by the action of ethyl chloroformate^76^ and transformation of the ester groups of (protoporphyrinato dimethyl ester)cobalt(II) to acyl azides using hydrazine and nitrous acid.[72a]

**Scheme 10:**
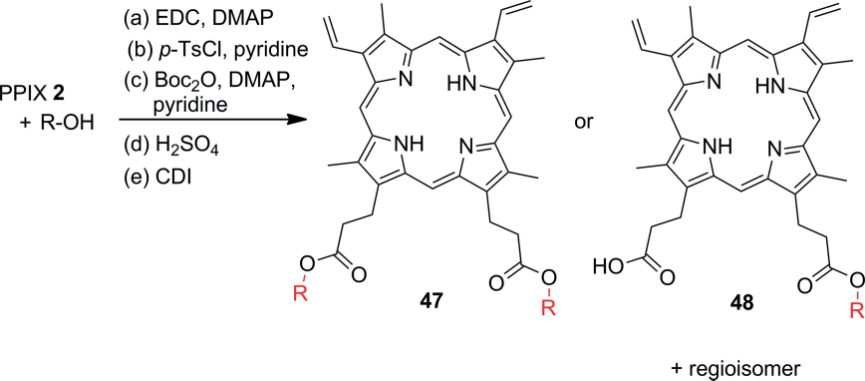
Strategies for ester bond formation at the PPIX carboxylic acid moieties.

The formation of different PPIX and hemin esters is also frequently used as a method to produce derivatives with certain new functionalities.^10^, [33b], 40 Examples are the incorporation of PPIX into polymeric materials, some of them with potential application in antimicrobial PDT,[68c], 77 synthesis of porphyrins with altered physicochemical properties^78^ and the preparation of a PPIX‐cobyrinate dyad as an activator of soluble guanylyl cyclase.^79^ Ester formation on PPIX was achieved using EDC and DMAP[77a], [77b], 79 or CDI[68c] and the respective hydroxyl‐substituted substrate to yield mono‐ and diesters **47** and **48**. Alternatively, esterification of PPIX acid moieties and cellulose hydroxyl groups was achieved by treatment with *p*‐toluenesulfonyl chloride (*p*‐TsCl) in pyridine (Scheme 10).[77c]

No additional activating agent was needed with hemin as cross‐linker in an epoxy resin curing reaction – the Fe(III) centre of hemin self‐catalyzed the reaction of the carboxylic acid moieties with the oxirane group of epoxy monomers to form polymeric structures[77d] Monoprotection of PPIX with *tert*‐butanol was achieved by activating one acid moiety with an equimolar amount of di‐*tert*‐butyl dicarbonate (Boc_2_O) and DMAP in pyridine, leaving one carboxylic acid moiety free for a subsequent peptide coupling reaction (Scheme 10).[72b], 80 Traditional sulfuric acid‐catalyzed esterification can be carried out when the respective alcohol is used as a solvent. In this manner, de Oliveira and co‐workers obtained PPIX derivatives with long alkyl substituents showing altered photophysical properties and aggregation behavior in non‐polar solvents.^78^

**Scheme 11:**
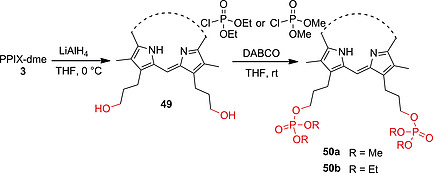
Reduction of ester groups to alcohols and phosphorylation.

The reduction of the dimethyl ester moieties of **3** with LiAlH_4_ leads to the formation of propyl alcohol side chains (**49**) which were treated with dialkylchlorophosphate and TEA to produce water‐soluble PPIX derivatives **50a** and **50b** (Scheme 11).[30a], 81

## 3. PPIX Derivatives for Chemical Biology Applications

Due to the plethora of uses of PPIX and its derivatives for biological applications we decided to limit our discourse to three main topics: sensors based on PPIX‐DNA complexes, the reconstitution of hemoproteins with PPIX derivatives and the use of metallo‐PPIXs as antimicrobial agents. Thereby, we discuss metallo‐ and free base porphyrins that are different from native heme whilst deriving from PPIX and retaining the important structural features of this porphyrin skeleton. The focus is on developments in the field that have taken place since 2006.

### 3.1 PPIX‐DNA‐Complex Sensing Devices

**Figure 2:**
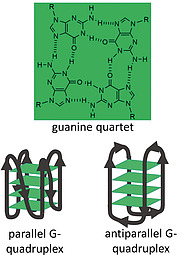
Hydrogen bonding in a guanine quartet and schematic representation of parallel and antiparallel G‐quadruplex conformations.

Guanine‐rich nucleic acid‐segments form particular secondary structures in vivo which function as regulators of genome and telomere stability as well as gene activity.[18a], 82 In such sequences the formation of guanine‐quartets (G‐quartets) via Hoogsteen base pairing can lead to the arrangement of the respective DNA or RNA sequences in four‐stranded helices, the so‐called G‐quadruplexes (Figure 2).^83^ Together with other molecules, these motifs have drawn attention as biochemical tools for in vitro applications such as DNAzymes to catalyze redox reactions in a peroxidase‐like fashion, as well as for the detection of enzymatic activity, nucleic acids, proteins, small molecules and metal ions.^83^, ^84^

The conformation of the G‐quadruplex is strongly dependent on the presence of certain metal ions interacting with the carbonyl oxygen atoms of the guanine bases inside the quartet. E.g., it was reported that binding to K^+^ ions leads to the formation of the most stable quadruplex structures and that the antiparallel conformation is especially stabilized. In contrast, Na^+^‐stabilized G‐quadruplexes preferentially adopt a parallel conformation of the strands (Figure 2).[83a], 85 Moreover, the G‐quartet constitutes a plane of aromatic moieties that can interact with aromatic molecules via π‐π‐interactions. Such interactions can be used to detect G‐quadruplex formation or to form functional entities such as DNAzymes. Among the compounds with G‐quadruplex binding abilities are tetrapyrroles such as phthalocyanines and porphyrins.[18a], 86 Notably, hemin binding for generation of DNAzymes with peroxidase‐like activity and for the development of molecular sensing devices has found widespread attention and has been discussed in several reviews.^83^, ^84^, [86a], 87 Herein, we will focus on the use of PPIX and its Zn(II) complex in conjunction with G‐quadruplex structures as biosensors.

The hydrophobic character of PPIX facilitates the formation of micellar aggregates in aqueous solution which quenches the molecules' fluorescence.^88^ Disruption of the aggregates by binding to the G‐quadruplex enhances the fluorescence of PPIX by a factor of 16. PPIX is reported to selectively bind to parallel G‐quadruplex conformers.[18a], 89 Zn(II)PPIX also shows a significant increase in fluorescence upon G‐quadruplex binding.^90^ Hence, both molecules can be used as fluorogenic reporters for G‐quadruplex formation which finds application for various sensing purposes that will be introduced hereafter.

#### 3.1.1 Detection of Nucleic Acids

**Figure 3:**
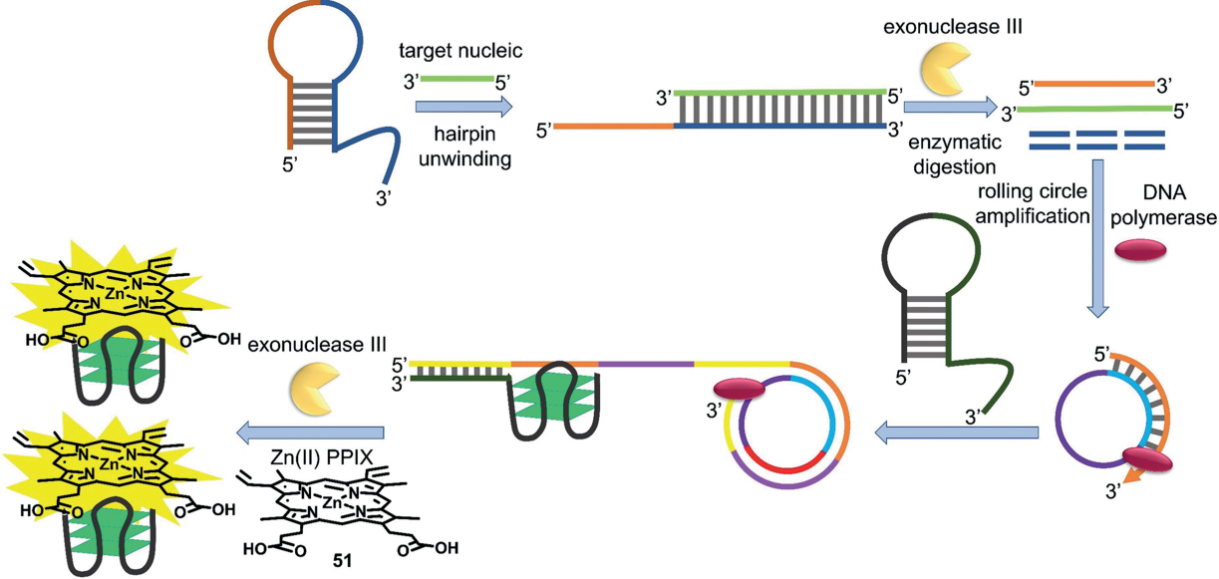
Exemplary nucleic acid detection with Zn(II)PPIX G‐quadruplex biosensors. Binding of the target nucleic acid unwinds the DNA hairpin. Subsequent digestion by exonuclease III liberates a DNA single‐strand that acts as a primer for rolling circle amplification. Binding of a G‐rich hairpin to the amplified DNA sequence causes refolding of the hairpin to a G‐quadruplex structure. Liberation of G‐quadruplexes by exonucleases and binding of Zn(II)PPIX (**51**) results in a fluorescence signal.

G‐quadruplexes in combination with PPIX have commonly been used for nucleic acid detection in vitro; e.g., for miRNA and DNA.[18b], [18c], 91 A frequently applied approach is the use of a DNA hairpin structure that unwinds following a binding event with the complementary target DNA or miRNA strand. The opening of the hairpin can be succeeded by digestion of the complex by appropriate exonucleases or another strand displacement event. This releases a single‐stranded DNA sequence, which recruits a guanine‐rich DNA strand that ultimately forms a G‐quadruplex structure. The process commonly involves a signal amplification cascade such as rolling circle amplification. Formation of the quadruplex can be detected by non‐covalent binding of surrounding PPIX or (protoporphyrinato IX)zinc(II) (Zn(II)PPIX, **51**) molecules, which results in an enhanced fluorescence signal (Figure 3).[18c], [91b], [91d]

Alternatively, the hairpin binding the target nucleic acid already contains guanine‐rich sequences which, after the opening of the hairpin, fold into a G‐quadruplex structure that induces a fluorescence signal upon porphyrin binding.[18b], [91c] Another approach taken by Yuan et al. involved unwinding of coiled single‐stranded DNA by binding to the target miRNA and used PPIX as fluorescent probe. This initial hybridization event induced rolling circle amplification of DNA which formed G‐quadruplex structures via a multistep cascade.[91a] A different strategy, applied for the detection of DNA as well as ATP, involved a three‐way DNA junction. Binding of the target DNA triggered a cascade which ultimately led to the recruitment of two DNA strands, each of them containing a part of a G‐quadruplex‐forming sequence. Bringing the two strands of this so‐called split G‐quadruplex in close vicinity to each other induced formation of the G‐quadruplex, followed by PPIX binding and a fluorescence signal.[91e] A split G‐quadruplex/PPIX system was also successfully applied by Wang et al. for the measurement of the length of DNA segments. The target DNA strand contained two sequences for hybridization of the two strands of the split G‐quadruplex. The larger the gap between the two sequences on the target strand the less efficient was the G‐quadruplex assembly and the lower the PPIX fluorescence. This method could be used for detection of a DNA length difference of as short as one base.[91f]

#### 3.1.2 Detection of Enzymatic Activity

G‐quadruplexes can be used to detect the activity of enzymes, e.g., for monitoring telomerase activity.^92^ Maintenance of telomeres, the terminals of chromosomes in human cells, by telomerase is usually inhibited in normal cells, while telomerase is active in the majority of tumor cells.^93^ Therefore, telomerase activity serves as a marker for the development of malignancies. Telomeres contain TTAGGG repeat sequences; thus, they form G‐quadruplex structures themselves. Colorimetric assays with hemin exploiting the peroxidase‐like activity of telomeric G‐quadruplexes have been used to detect telomerase activity.^92^ A now classic system by Willner and co‐workers quantified the activity of telomerase in cancer cell lysates based on the fluorescence of Zn(II)PPIX. A telomerase primer served as a substrate to be telomerized by the enzyme from the cell extracts and G‐quadruplex formation in the so‐obtained telomere repeats was detected by Zn(II)PPIX binding and its fluorescence response.[91c]

The activities of other DNA‐editing and ‐degrading enzymes have been monitored by using a combination of G‐quadruplexes and PPIX or Zn(II)PPIX.^94^ Detection of DNA methylation patterns, an epigenetic modification accomplished by DNA methyltransferases, is also a means of early cancer recognition.^95^ Methylation of adenines in the sequence of a DNA hairpin probe led to the recognition of the methylated sites by endonuclease Dpn1 and the liberation of single‐stranded DNA. After undergoing an amplification cascade, G‐quadruplexes were recruited that bound (protoporphyrinato IX)zinc(II).[94a], [94b]

Detection of the activity of uracil glycosylase, an enzyme involved in base excision repair of DNA, was carried out similarly. Uracil excision in one strand of a DNA duplex liberated the complementary strand which subsequently acted as a primer for rolling circle amplification. The so‐formed G‐rich DNA strand folded into G‐quadruplex structures that were fluorescently detected by PPIX binding.[94d]

Zhou et al. accomplished monitoring of the activity of the DNA‐digesting enzyme endonuclease S1 using a G‐quadruplex/PPIX‐based sensor. The intact G‐quadruplex assembly gave a high fluorescence readout, while the degradation of G‐quadruplex‐DNA by endonuclease S1 led to a decrease in PPIX fluorescence. The presence of K^+^ ions inhibited the digestion of G‐quadruplexes by the endonuclease, making the system not only feasible as a sensor for enzyme activity but also as a K^+^ sensor.[94c]

#### 3.1.3 Detection of Metal Ions

The inherent property of G‐quadruplexes to take on different conformations of different stabilities upon binding to various metal ions has been used for fluorescent detection of metals with PPIX as a signal reporter.[83a], [85b], 89, 90, [94c], 96 For example, Li et al. analyzed the ability of PPIX to bind to the G‐quadruplex‐forming DNA sequence PS2.M and showed that PPIX preferentially binds parallel G‐quadruplex structures with an about 100‐fold selectivity over duplexes and antiparallel G‐quadruplexes. DNA binding resulted in a 10‐fold increase in the porphyrin fluorescence. This enabled monitoring of DNA structural changes upon changing from Na^+^‐stabilized antiparallel to K^+^‐stabilized parallel G‐quadruplexes and suggests the use of PPIX/G‐quadruplex systems as K^+^ sensors.^89^ Ma et al. investigated the competitive binding of K^+^ and Na^+^ to G‐quadruplex‐forming DNA sequence PW17 in more depth and discovered that K^+^ can replace Na^+^ in PW17 to form a K^+^‐stabilized parallel G‐quadruplex. The transition passes through a stable intermediate binding a K^+^ and a Na^+^ ion in one G‐quartet. K^+^ detection in the presence of Na^+^ was accomplished using PPIX as fluorescent read‐out probe.[85b]

In addition to the binding of K^+^ and Na^+^, G‐quadruplexes interact with other metals, a property that has found application for heavy metal detection. E.g., Li et al. showed that Pb^2+^ can compete with K^+^ for binding to PW17 and PS2.M G‐quadruplexes. The Pb^2+^‐stabilized G‐quadruplexes exhibit an antiparallel conformation and the structural change from parallel to antiparallel was detected via hemin and Zn(II)PPIX release from the DNA, resulting in a loss of DNAzyme activity or fluorescence decrease, respectively.^90^ The ability of Pb^2+^ to efficiently stabilize G‐quadruplexes was later exploited to design a Pb^2+^ sensor based on the Pb^2+^‐induced transition of a DNA molecule from its duplex to a quadruplex conformation via liberation of a DNA single strand. Specifically, the G‐quadruplex used, T30695, bound Zn(II)PPIX under the significant enhancement of the porphyrin's fluorescence and the sensor could be rendered reversible by addition of a Pb^2+^ chelator.[96d] The T30695 G‐quadruplex was also used for the detection of Pb^2+^ in the presence of Na^+^. It was found that Na^+^ induces conformational changes of the Pb^2+^‐stabilized G‐quadruplex from partly to completely parallel, resulting in a more efficient binding of Zn(II)PPIX as the fluorescent probe.[96a]

A complementary detection strategy is the disruption of stable G‐quadruplexes by destabilizing metal ions. In this manner, Hg^2+^ ions were detected using a G‐quadruplex‐forming DNA sequence containing six thymine bases. The K^+^‐stabilized G‐quadruplex binds PPIX, thereby enhancing its fluorescence. The presence of Hg^2+^ induced transition of the quadruplex to a hairpin‐like DNA duplex, causing unbinding of the fluorescent probe and decrease of the signal.[96b]

A yet different strategy was applied for the detection of Cu^2+^ ions using a PPIX/G‐quadruplex system. G‐quadruplex‐based DNAzymes can catalyze the insertion of Cu(II) and Zn(II) into mesoporphyrin IX^97^ This finding was implemented by Wang and co‐workers who developed a Cu^2+^ sensor based on G‐quadruplex‐catalyzed metalation of PPIX. Upon Cu(II) insertion the fluorescence of the PPIX/G‐quadruplex was quenched.[96c]

#### 3.1.4 Detection of Small Molecules and Proteins

G‐quadruplex‐based sensing devices have also been used for the detection of small molecules,[91c], [91e], 98 proteins^99^ and cells.^100^ The nucleotide ATP was detected using an aptamer‐forming DNA sequence that only assembled to a G‐quadruplex structure upon ATP binding. Zn(II)PPIX binding gave a fluorescent indication of the DNA structural change.[91c] A different strategy involved ATP binding and aptamer folding with one of the DNA strands in a three‐way DNA junction which induced displacement of the strand. This prevented the downstream cascade of G‐quadruplex formation and PPIX binding from taking place.[91e]

A similar method was used for the detection of the mycotoxin ochratoxin A. G‐quadruplex formation upon binding of ochratoxin A to the DNA aptamer and recruitment of Zn(II)PPIX effected the fluorescent signal readout.[98a] A local surface plasmon resonance (LSPR)‐based ochratoxin sensor was designed that included DNA‐aptamers immobilized on LSPR‐active gold nanorods with Zn(II)PPIX again acting as signal enhancer. The aptamers were used to bring the plasmonic sensor and the analyte closer together, thereby improving the signal intensity. Nevertheless, Zn(II)PPIX was found to give a lower fluorescent readout than other signal reporters, such as berberine, and was not employed further in this sensing system.[98b]

A protein‐binding DNA aptamer in combination with a G‐quadruplex was used for thrombin detection. Thrombin binding at the 3'‐terminus of a DNA aptamer sequence induced folding of the latter protecting the oligonucleotide from degradation by exonuclease I. Subsequently, a guanine‐rich DNA hairpin was hybridized to the thrombin‐bound oligonucleotide under opening of the hairpin. G‐quadruplex formation of the guanine‐rich sequence and Zn(II)PPIX binding resulted in a fluorescent response.^99^

Porphyrins themselves can also be detected with such systems. Competitive binding of PPIX and hemin to a G‐quadruplex structure was used for hemin detection. Hemin replaced PPIX bound to G‐quadruplex, thereby quenching the fluorescence of the system.[98c]

Lastly, G‐quadruplex systems were used for the cellular delivery of PPIX as a photosensitizer and fluorescent probe.^100^ By binding to the G‐quadruplex the low solubility of PPIX in an aqueous medium can be overcome. More importantly, the G‐quadruplex aptamers can be designed to bind to specific cellular proteins, which can mediate cell and cell organelle targeting. This way, PPIX/G‐quadruplexes can act as cancer therapeutics in PDT and cancer cell imaging tools. Wang and co‐workers reported that nucleolin, a protein that is overexpressed in cancer cells, can act as a PPIX/G‐quadruplex target and can impart binding of this system on the cell surface[100a] as well its internalization.[100b]

### 3.2 Reconstitution of Hemoproteins with Non‐Natural Cofactors

The reconstitution of hemoproteins with non‐natural metallo‐porphyrin cofactors is a potent tool to tweak enzymes to perform specific chemical reactions in vitro. While the metal complexes are the catalytically active part, the protein provides the scaffold for substrate binding, macrocycle conformational control, and orientation relative to the metal cofactor; thus, it impacts the activity and selectivity of the reaction. Modulation of chemical transformations by metallo‐enzymes, therefore, can be achieved by alteration of the metal center, its ligand, and the protein environment.^101^

In exemplary work, Marletta and co‐workers explored different ways of modulating hemoproteins from the heme nitric oxide/oxygen binding domain (H‐NOX) family to produce proteins with altered functions. Engineering of the heme binding pocket of the protein by mutagenesis as well as replacement of the native heme cofactor with Ru(II) mesoporphyrin IX were employed to modulate the NO and O_2_ binding abilities of H‐NOX rendering it a potential sensor for these gases.^102^ Stable complexes of H‐NOX proteins with the paramagnetic heme analogues Mn(II)/(III)PPIX and Gd(III)PPIX were assessed as magnetic resonance imaging (MRI) contrast agents that do not suffer from leaching of toxic metal into the tissue.^103^ A different tool used by Nierth and Marletta for the modification of H‐NOX hemoproteins was synthetic modification of the porphyrin scaffold. An alkynyl moiety was introduced in one of the meso‐positions of heme. After reconstitution of H‐NOX with the modified heme cofactor, this left a synthetic handle for attachment of fluorophores or groups that alter the redox potential of the cofactor.^104^ These key advancements illustrate the potential of cofactor reconstitution in hemoproteins as a tool to produce biomolecules with altered or enhanced functionalities.

#### 3.2.1 Oxidation/Reduction Catalysts

**Scheme 12:**
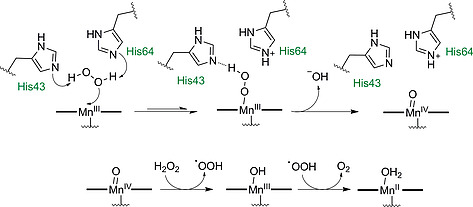
Proposed mechanism for H_2_O_2_ splitting in a Mn(III)PPIX reconstituted F43H myoglobin mutant, assisted by amino acid residues His43 and His64.

**Scheme 13:**
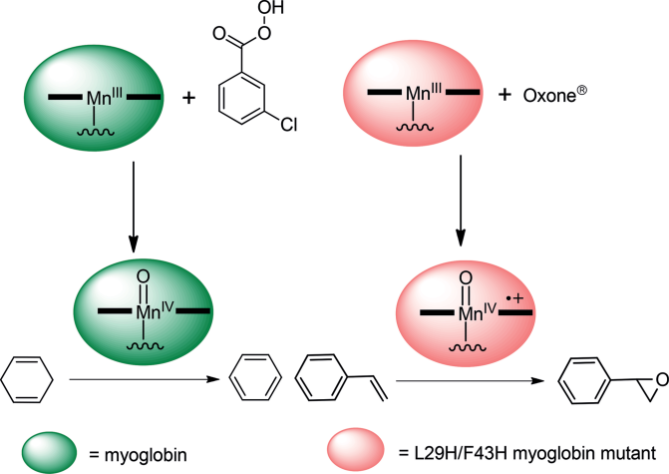
Oxidation of 1,4‐cyclohexadiene to benzene with *m*CPBA and oxidation of styrene to 2‐phenyloxirane with oxone catalyzed by Mn(III)PPIX reconstituted myoglobin and the L29H/F43H mutant of myoglobin, respectively.

Next, we focus on the incorporation of non‐heme metallo‐protoporphyrin complexes into protein matrixes and their use as biohybrid catalysts. Nature employs heme‐dependent enzymes such as catalase, cytochrome P450 enzymes, nitric oxide synthase, and peroxidases to catalyze redox reactions.[5b] Replacing iron by other metals can help to elucidate the mechanism of action of heme enzymes and can also yield oxidizing/reducing enzymes with altered catalytic activity and substrate scope. One promising candidate for hemoprotein reconstitution is Mn‐PPIX. It has been used to replace heme in different proteins and these constructs have been evaluated for their peroxidase‐like activity. H_2_O_2_ cleavage by Mn(III) enzymes to give O_2_ and H_2_O passes through H_2_O_2_ binding to the metal center and oxidation to high valent Mn‐oxo complexes Mn(IV)=O or Mn(V)=O, a process equivalent to native heme‐based peroxidases. However, (protoporphyrinato)manganese(III) (Mn(III)PPIX) reconstituted proteins were shown to be less efficient in cleaving peroxides compared to their native iron‐containing counterparts.^105^ Zhang and co‐workers successfully modulated the peroxidase‐like activity of Mn(III)PPIX reconstituted myoglobin by altering the protein scaffold. Amino acid residue replacements showed that His64 in the wild‐type protein was crucial for enzymatic activity. This was imparted by hydrogen bond formation between His64 and H_2_O_2_, thereby facilitating H_2_O_2_ binding to the Mn(III) center. The additional introduction of the residue His43 led to a 5‐fold increase in H_2_O_2_ activation by the enzyme, probably by the formation of an additional hydrogen bond between His43 and H_2_O_2_ and facilitated O–O bond cleavage (Scheme 12).^106^

(Protoporphyrinato)manganese(III) reconstituted proteins were used to catalyze the oxidation of organic compounds.^107^ A Mn(IV)‐oxo complex was produced from Mn(III)PPIX myoglobin by addition of the two‐electron oxidant *meta*‐chloroperbenzoic acid (*m*CPBA) (Scheme 13). This oxidative Mn(IV) intermediate was capable of catalyzing C–H bond cleavage in 1,4‐cyclohexadiene yielding benzene, a reaction that could not be catalyzed by native myoglobin.[107a] An engineered Mn(III)PPIX myoglobin in which the amino acid residues Leu29 and Phe43 had been replaced with His was successfully used for the epoxidation of the styrene double bond using oxone as an oxidant in place of H_2_O_2_. For oxone, the peroxide coordinated to the Mn(III) center undergoes heterolytic bond cleavage giving a Mn‐oxo radical cation intermediate [Mn(IV)=O]^**·**+^ (Scheme 13). This complex is far more reactive in oxidation reactions than the Mn(IV)=O complex formed by homolytic peroxide bond cleavage with H_2_O_2_. The His residues in the positions 29, 43 and 64 provide the protein coordination sphere necessary for orientation and activation of the oxidant.[107b]

Mixed oxidation state Os(III)/(VI)‐PPIX in a horseradish peroxidase (HRP) scaffold was also tested for its ability to reduce hydrogen peroxide. The reconstituted enzyme immobilized on a glassy carbon electrode showed less propensity for the electrocatalytic reduction of H_2_O_2_ compared to native HRP but higher efficiency for the reduction of *tert*‐butyl hydroperoxide. This suggests Os‐PPIX HRP electrodes as possible biosensors for organic peroxides.^108^

**Figure 4:**
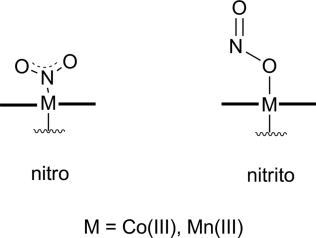
Nitro and nitrito modes of nitrite binding to Co(III)‐ and Mn(III)PPIX reconstituted myoglobin.

Metallo‐PPIX reconstituted myoglobins were used as models for the nitrite reductase activity of hemoproteins. E.g., deoxy‐hemoglobin and deoxy‐myoglobin were found to convert nitrite to the versatile physiological signaling molecule NO.^109^ Richter‐Addo, Ford and co‐workers studied crystal structures of Mn(III)‐ and Co(III)PPIX myoglobins where the metal centers were coordinated with different small molecule ligands, including nitrite. An oxygen‐bound nitrito coordination mode was found for both, Mn(III) and Co(III) metal centers, which was mediated by hydrogen bonding between nitrite and distal residue His64 (Figure 4). This residue is also responsible for hydrogen bonding of the NO ligand in the reduced Mn(II) form of the enzyme.^110^ Rate constants of nitrite reduction by the reduced forms, Mn(II) and Co(II) myoglobin, were studied and both complexes exhibited nitrite reductase activity; however, with much lower efficiency compared to wild‐type ferrous myoglobin. In the case of Co(II) myoglobin, this was attributed to it being a poorer reductant than Fe(II) myoglobin, whereas in the case of Mn(II) myoglobin other factors were predicted to be critical such as the stability of the metal‐NO complexes.^111^

Mn(III)PPIX^112^ in combination with genetically engineered human serum albumin (HSA) was also employed as an artificial superoxide dismutase. Wild‐type HSA Mn(III)PPIX showed only little activity in dismutating the reactive superoxide radical anion O_2_^**·**–^, whereas when the proximal amino acid residue Tyr161 was replaced by Leu via site‐directed mutagenesis, the catalytic activity increased by a factor of 5.5. Presumably, this was due to the removal of Tyr161 as an axial ligand for Mn(III)PPIX and exchange with a non‐coordinating residue. This led to the formation of a superoxide dismutase where the metalloporphyrin plane and catalytic cavity are not blocked by a coordinating amino acid residue.[20c]

**Figure 5:**
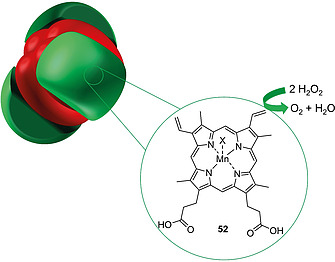
Schematic representation of a protein cluster consisting of three Mn(III)PPIX^113^ (**52**) reconstituted HSA units (green) wrapped covalently around hemoglobin (red). The HSA units feature catalase activity.

Upon mutation of Tyr161 in HSA to a His residue instead and incorporation of Mn(III)PPIX (**52**) into the binding pocket, the protein showed catalase activity. This ability of Mn(III)PPIX‐HSA to catalyze the degradation of H_2_O_2_ was exploited by covalently conjugating it to hemoglobin (Figure 5). In this cluster, the engineered HSA acted as a protective wrap for hemoglobin, which is prone to oxidation by self‐produced and surrounding H_2_O_2_. Thereby, hemoglobin was protected from oxidation by H_2_O_2_ which renders the protein‐hybrid promising for use as a hemoglobin‐based O_2_ carrier (HBOC), e.g., for therapeutics.^113^

Jones and co‐workers studied the influence of the metallo‐PPIX cofactor on the redox potential and electron transfer properties of the bacterial hemoprotein cytochrome b_562_. While the protein bound to Zn(II)PPIX did not show any redox activity, as was expected, the incorporation of Cu(II)PPIX shifted the redox potential by +300 mV as compared to the native heme enzyme. The Cu‐reconstituted protein also showed substantial electron transfer abilities. These findings underline the idea that non‐native metalloporphyrin cofactors can be used to tune the redox chemistry of metallo‐enzymes.^114^

#### 3.2.2 Carbene Insertion Catalysts

Engineered and natural hemoproteins are highly promising catalysts for abiological reactions, especially C–H bond activation reactions. Among those are N–H, and S–H insertions, as well as cyclopropanations and aziridinations.[20a], [20b] However, the use of heme as a co‐factor in abiological catalysis restricts the applicability to a limited set of reactions. Considering the importance of transition metal catalysis in modern synthetic chemistry, the replacement of heme‐iron by different transition metals could lead to a drastic expansion of the scope of accessible “heme”‐enzyme‐catalyzed reactions. Hence, reconstitution of heme enzymes with metallo‐PPIX complexes to catalyze chemical transformations of C–H bonds in small molecules has been attempted.^115^

A (protoporphyrinato)manganese(III) chloride [Mn(III)PPIX(Cl)] myoglobin complex was tested for its catalytic activity towards the hydroxylation of ethylbenzene to 1‐phenylethanol, but was found to be inactive. Only the Mn(III)porphycene(Cl) analogue showed efficacy in catalyzing the reaction.[115a] Mn(II) and Co(II)PPIX myoglobin complexes were shown to catalyze intramolecular C–H amination reactions of arylsulfonyl azides. Engineering the protein active site by point mutations resulted in enhanced stereo‐ and enantioselectivity in ring closure reactions of the substrate. The substrate total turnover numbers of both, Mn(II)‐ and Co(II)porphyrin reconstituted myoglobins were found to be lower (142 and 64, respectively) compared to wild‐type myoglobin (181).[115b]

**Figure 6:**
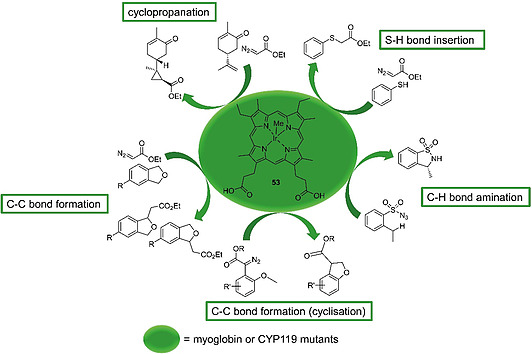
C–C bond formation, cyclopropanation S–H bond insertion and C–H bond amination reactions catalyzed by (methyl)(mesoporphyrinato IX)iridium(III) [Ir(III)MPIX(Me)] (**53**) reconstituted myoglobin or CYP119 mutants, respectively.

Hartwig and co‐workers systematically screened a library of myoglobins reconstituted with different metallo‐PPIX and metallo‐mesoporphyrin IX cofactors for their catalytic activity in C–C bond forming reactions which were not catalyzed by wild‐type myoglobin. Two model reactions were chosen to test the different metalloproteins: an intramolecular carbene insertion into a C–H bond and cycloadditions of carbenes to alkenes (cyclopropanation). The best turnover numbers for both reactions (>60) were obtained using axially methyl‐ligated iridium complex (methyl)(mesoporphyrinato IX)iridium(III) [Ir(III)MPIX(Me)] (**53**) as cofactor (Figure 6). A stepwise evolution of the protein scaffold was carried out in order to optimize the stereoselectivities of both reactions. Exchanging different amino acids in the inner and outer coordination sphere of the enzyme resulted in good to high *er* and *dr* values for formation of the asymmetric products; yet, the substrate turnover numbers were low.^116^

To improve substrate conversion rates, a cytochrome P450 hemoprotein (CYP119) from a thermophilic archaeon was employed as a protein scaffold instead of myoglobin. The thermostable CYP119 enzyme is involved in stereoselective biosynthetic transformations of C–H bonds in its wild‐type form. Reconstitution of CYP119 with Ir(III)MPIX(Me) (**53**) and directed evolution of the apoprotein to enhance the substrate affinity yielded an enzyme with vastly increased efficiency in C–H carbene insertion reactions; more than 4000 times higher than that of the wild‐type heme enzyme. The artificial enzyme catalysed reactions with turnover numbers up to 35 000 and up to 94 % *ee*.^117^ The Ir(III)MPIX(Me)–CYP119 system was also adapted for the catalysis of stereoselective cyclopropanation reactions for a variety of olefinic substrates (Figure 6).^118^

Intramolecular C–H bond amination of sulfonyl azides, which had been attempted previously with rather low efficiencies using Mn(II)‐ and Co(II)PPIX‐reconstituted myoglobin,[115b] was also revisited in light of these discoveries. CYP119 was reconstituted with different PPIX and mesoporphyrin IX metal complexes. Again, Ir(III)MPIX(Me) proved to be the most suitable enzyme cofactor giving the highest chemoselectivities and good yields (Figure 6). Screening of metalloporphyrin reconstituted CYP119 variants produced by site‐directed mutagenesis revealed mutants that catalyzed the reaction with increased turnover numbers (≈300) and high *er* values (95:5).^119^ The scope of reactions catalyzed by engineered Ir(III)MPIX(Me)‐CYP119 enzymes was expanded further to the insertion of carbenes generated from ethyl 2‐diazoacetate into C–H bonds of various 4‐substituted phthalanes (Figure 6).^120^ Fasan and co‐workers used such an approach with different metallo‐PPIX and ‐MPIX complexes for carbene insertion reactions into N–H, S–H, and C–H bonds, as well as cyclopropanations. For N–H insertion, myoglobin variants containing heme as a cofactor showed the highest turnover numbers and product yields. The same was found for cyclopropanation reactions. S–H and C–H insertions were most efficiently catalyzed by engineered Ir(III)MPIX(Me)‐myoglobin (Figure 6).^121^

Lehnert and co‐workers engineered myoglobin by replacing the proximal His64 in the protein with different amino acid resides and reconstituted the apoprotein with Ru(II)MPIX^124^ Although Ru complexes were assessed as promising catalysts for the N–H insertion and cyclopropanation reactions, the Ru(II)MPIX‐myoglobin variants tested showed low activities in these reactions. It was suggested that catalytic activity could be greatly improved by further mutations in the protein's active site^122^ as it had been demonstrated previously by Sreenilayam and Fasan for heme‐bound myoglobin.^123^

In parallel Brustad's group engineered the heme binding pocket in a cytochrome P450 from *Bacillus megaterium* to enable the protein to distinguish between the native PPIX and the smaller deuteroporphyrin IX (DPIX) scaffolds.^124^ Selective incorporation of Ir(III)DPIX(Me) into engineered cytochrome P450 variants produced catalysts for cyclopropanation reactions for which the native enzyme was inactive. Reactions were catalyzed with relatively low turnover numbers (up to 147) and moderate diastereoselectivity (13:87).[124b]

#### 3.2.3 Construction of Photosensitive Proteins

Embedding metallo‐PPIX photosensitizers in a protein matrix prevents aggregation‐induced quenching of their excited states and additionally allows for tuning of the sensitizers' properties and their orientation towards electron‐accepting substrates, e.g., by modulating the coordination sphere of the protein.

Previously, it has been shown that PPIX bound inside a protein matrix can act as a photosensitizer and that it reacts with singlet oxygen and other reactive species produced in the sensitization process to form photoadducts.^125^ The porphyrin can also induce a conformational change in the protein it is bound to. This was shown with PPIX embedded in *β*‐lactoglobulin, a protein that is not a native ligand for this dye. Irradiation of the porphyrin at 405 nm caused a local unfolding of the protein; this was not induced by singlet oxygen but was assumed to arise from electron transfer events from PPIX to surrounding amino acids.^126^ The generation of singlet oxygen inside the protein matrix of Zn(II)PPIX reconstituted myoglobin was studied by Lepeshkevich et al. Although the metalloprotein produced singlet oxygen, the production efficiency was reduced by a lower frequency of collisions between molecular oxygen and triplet state Zn(II)PPIX as well as the non‐radiative deactivation of singlet oxygen by collisions with amino acids.[24c]

**Figure 7:**
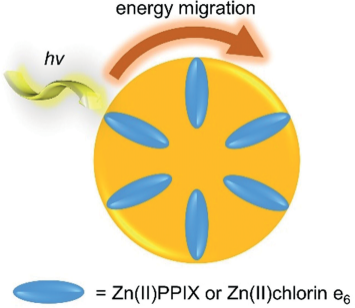
Hexameric heme protein reconstituted with Zn(II)PPIX and Zn(II)chlorin e_6_ for studies of energy migration in the complex.

Photosensitive proteins are often used in assemblies as models for native light‐harvesting complexes. In the present context, a hexameric heme protein isolated from a marine bacterium was reconstituted with the photosensitizers Zn(II)PPIX and Zn(II)chlorin e_6_ [(15‐carboxymethyl‐3^1^,3^2^‐didehydro‐rhdochlorinato)zinc(II)]. Each of the six subunits bound one tetrapyrrole metal complex, leading to the arrangement of the photosensitizers in a spatially and conformationally defined manner (Figure 7). After irradiation, the energy migration between the subunits of the complex was studied. The researchers found an indication that singlet‐singlet annihilation takes place in the oligomeric hemoprotein.[24b]

**Figure 8:**
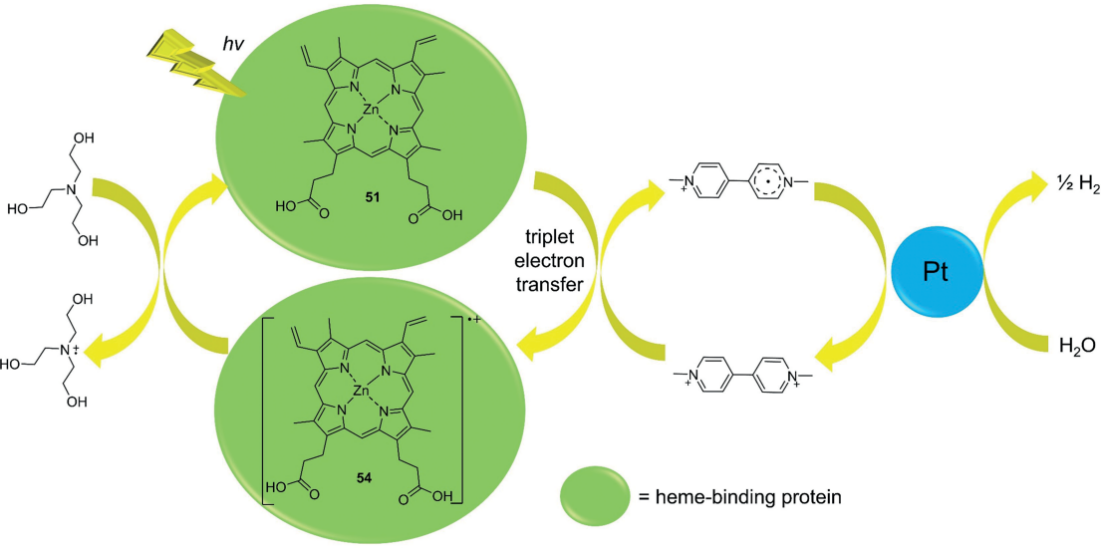
Schematic representation of photosensitized water reduction with a Zn(II)PPIX (**51**) reconstituted heme‐binding protein as the photosensitizer, 1‐methyl‐4,4'‐bipyridinium (MMBP^+^) as the electron mediator, colloidal Pt as the reduction catalyst and triethanolamine as the sacrificial electron donor.

Komadsu, Tsuchida, and co‐workers were the first ones to use a Zn(II)PPIX‐based protein complex for photosensitized water reduction. For this Zn(II)PPIX (**51**) was incorporated into HSA and the construct was combined with the electron mediator 1,1'‐dimethyl‐4,4'‐bipyridinium (DMBP^2+^), colloidal Pt‐polyvinyl alcohol (PVA) particles as an electron transfer catalyst, and triethanolamine as a sacrificial electron donor for restoration of the photosensitizer (Figure 8). Photoinduced electron transfer (PET) was found to take place only via the triplet state pathway of the photosensitizer to form radical cation **54**. The system showed good efficiency for photosensitized reduction of water to hydrogen when compared to the (5,10,15,20‐tetrakis(*N*‐methylpyridinium‐4‐yl)porphyrinato)zinc(II)‐HSA complex.^127^

Hayashi et al. also investigated the use of light‐sensitive proteins for catalytic processes. They reconstituted apomyoglobin with Zn(II)PPIX that was modified at the carboxylic acid moieties to carry charged groups which can attract ionic electron acceptors via electrostatic interactions. The PET from the photosensitizer to the acceptor was studied.[42f], 128 Later, the researchers used such a complex of myoglobin reconstituted with a Zn(II)PPIX derivative carrying anionic moieties for the photocatalytic reduction of H_2_O to H_2_. They combined the photoactive protein with the cationic electron mediator 1‐methyl‐4,4'‐bipyridinium (MMBP^+^), a polyvinylpyrrolidone‐coated Pt‐colloid as a catalyst and triethanolamine as a sacrificial electron donor. Electron transfer from the photosensitizer was found to occur from its triplet excited state. The use of monocationic MMBP^+^ as opposed to the more commonly used dicationic DMBP^2+^ proved beneficial because the neutral MMBP^**·**^ formed after the PET event diffused more easily from the anionic porphyrin site reducing back electron transfer to the porphyrin.^129^

A similar approach was later used for the generation of a photocurrent induced by gold surface‐immobilized photosensitizer‐protein assemblies. Zn(II)PPIX derivatives, covalently modified to carry thiol arms, were immobilized on a gold surface. In parallel, apocytochrome b_562_ was covalently attached to another Zn(II)PPIX derivative via a Cys residue in the protein. Interactions of the vacant heme binding pocket of cytochrome with Zn(II)porphyrin bound to another cytochrome molecule led to the formation of protein rods. These were immobilized on the Zn(II)PPIX‐modified Au surface through protein‐porphyrin interaction, yielding a supramolecular assembly of photoactive proteins on the surface. Irradiation with monochromatic light generated a photocurrent which was significantly enhanced compared to a surface with a single layer of photoactive hemoproteins.[72b] Assembly of the metalloprotein rods with opposite orientation in regards to the gold electrode also resulted in photocurrent generation.^130^

Bacterioferritin (Bfr), a bacterial iron storage protein that can bind heme in its 24‐meric shape, was also employed as a protein scaffold for Zn(II)PPIX and Zn(II)MPIX photosensitizers. Genetically engineered Bfr dimers were expressed in which 10 amino acid residues at the porphyrin binding site had been replaced by Gly residues to facilitate access for redox‐active molecules and solvents. The dimers were found to bind one Zn(II)porphyrin each. 1:1 Mixtures of Zn(II)porphyrin‐Bfr dimers and Pt nanoparticles encapsulated by a Bfr 24‐mer were prepared. In the presence of DMBP^2+^ and triethanolamine the system showed light‐induced H_2_ generation that was comparable to similar clusters.^131^ Further development of a Bfr dimer with one Zn(II)PPIX unit bound in combination with a colloidal Pt nanoparticle catalyst and an ascorbate‐containing buffer as sacrificial electron donor showed superior H_2_ production in water than the previously reported system. Remarkably, no electron transfer mediator was needed for the reaction.^132^

Co‐PPIX^133^ reconstituted myoglobin was also used for photoinduced hydrogen production in water. The system consisted of the protein‐porphyrin complex, Ru(Bpy)_3_^2+^ and sodium ascorbate as a sacrificial electron donor. Irradiation with visible light under aerobic conditions triggered proton‐coupled electron transfer from the metalloprotein resulting in hydrogen production. The Co(I)/Co(0)PPIX‐myoglobin redox couple was assessed as the catalytically active species. The protein assembly showed a 3‐fold higher turnover number than free Co‐PPIX.^133^ The catalytic activity could be increased by altering the secondary coordination sphere of the protein using point mutations. E.g., the H64A and H97/64A mutants showed higher activity than the wild‐type myoglobin porphyrin complex.^134^

### 3.3 Antimicrobial Activity of PPIX Metal Complexes

In 1999, Srinivasan and co‐workers discovered that PPIX metal complexes containing non‐natural metals exhibit antibacterial activity against Gram‐positive, Gram‐negative as well as Mycobacteria.^135^ Bacterial growth inhibition by these compounds was attributed to their uptake into the bacterial cell, which either occurs through a “Trojan horse” mechanism or via direct penetration of the membrane. The Trojan horse pathway involves binding of the abiological porphyrins to proteins that are normally responsible for shuttling heme into the bacterial cells and “tricking” these proteins into transporting the non‐natural heme analogues instead. Once in the cell, the metallo‐protoporphyrins are assumed to cause cytotoxicity through various effects: inhibition of heme‐dependent enzymes, the release of cytotoxic metals, promotion of reactive oxygen species (ROS) generation and interference with iron‐dependent processes.^136^

Another mode of action is the inhibition of heme transport through the membrane by irreversible binding of metallo‐protoporphyrins IX to proteins involved in the bacterial heme uptake.[136b], 137 As pathogenic bacteria rely on the acquisition of iron from the host organism, these findings suggest the potential application of non‐natural PPIX metal complexes as antibiotics. Pathogenic bacteria sequester iron from their host organism, by uptake of either free metal ions or of heme. Thus, bacterial heme uptake can be exploited for the delivery of drugs into the bacterial cell or the heme transporting complex can be blocked to deprive the bacterium of its iron source.[136a]

Co(III)PPIX was shown to inhibit heme uptake in the Gram‐negative bacterium *Escherichia coli*. The non‐iron heme analogue was taken up into the bacterial cell; however, no cytotoxicity directly associated with it was detected. Heme levels in bacteria growing in Co(III)PPIX‐containing medium were lower than without Co(III)PPIX, showing that the analogue competes with heme for uptake into cells.^138^

The heme analogues In(III)‐, Ga(III)‐, Co(III)‐ and Cu(II)PPIX showed bactericidal activity against the Gram‐negative pathogen *Porphyromonas gingivalis*, owed to their toxicity after cell internalisation.^142^ The metalloporphyrins inhibited *P. gingivalis* biofilm formation and infection of epithelial cells.[139b] Further studies focused on the bacterium's hemophore HmuY revealed that Ga(III)‐, Zn(II)‐, Co(II)‐ and Mn(III)PPIX cannot be displaced from HmuY by heme due to formation of stable coordination complexes with distal His residues in the protein's heme pocket. In contrast, Cu(II)‐ and Ni(II)PPIX bind only weakly to the His134 residue in the hemophore and can, therefore, be replaced by heme. The blockage of HmuY by stable binding of non‐iron metallo‐porphyrins might contribute to their antibacterial effect.^140^

Heme uptake in the Gram‐positive pathogenic bacterium *Staphylococcus aureus* is dependent on nine proteins located in the membrane and the cell wall. These Isd proteins take on different positions in a heme transfer cascade. Non‐iron metallo‐porphyrins such as Ga(III)‐ and Mn(III)PPIX have been shown to effectively bind to Isd receptors and exhibited antibacterial activity towards *S. aureus*, whereas weaker Isd protein binders such as Mg(II)‐, Zn(II)‐ and Cu(II)PPIX showed less bactericidal effects.^141^ Binding of the heme analogues Co(III)PPIX, Fe(III)PPIX‐dme and Mn(III)PPIX to cell wall‐associated Isd proteins in *S. aureus* was analyzed using different spectroscopic techniques. While Mn(III)PPIX was transferred between the transport proteins of the cascade, Co(III)PPIX and Fe(III)PPIX‐dme were found to bind to specific Isd proteins and were not passed on to other proteins of the cascade. Thus, these compounds show potential as antimicrobial agents through inhibition of the Isd heme transport system, thereby starving or weakening the bacterium.^142^

Ga(III)‐ and Zn(II)PPIX were also shown to inhibit aerobic respiration in *S. aureus* via incorporation into the cytochromes of the bacterium's terminal oxidases, thereby disrupting the electron transport chain.^143^ Skaar and co‐workers suggested that Ga(III)‐, Mn(III)‐ and, to a lower extend, Zn(II)PPIX induced the heme detoxification system in *S. aureus*, a cluster of proteins which is responsible for efflux of excess heme out of the bacterial cell. Nevertheless, the transport proteins of the detoxification system were found to be inefficient in shuttling the non‐iron metalloporphyrins. On the contrary, the upregulation of the heme detoxification system induced by non‐iron metalloporphyrins resulted in lower viability of bacterial cells. Presumably this was attributed to the high energetic costs for the bacteria associated with the expression of the transport proteins.^144^

Ga(III)PPIX was also shown to inhibit the growth of *Mycobacterium abcessus*, a bacterial strain associated with infections in cystic fibrosis patients^145^ indicating that non‐iron metallo‐PPIXs could be used as anti‐mycobacterial agents.[136b] Similarly, Sn(II)PPIX and Pd(II)MPIX reduce cell growth of the protoazon parasite *Trypanosoma cruzi* by inhibition of its heme uptake system.^146^

## 4. Closing Remarks

Nature's porphyrin allrounder, PPIX, has made its way into a multitude of disciplines all across the natural sciences due to its inherent chemical and physical properties, ready availability, and relevance in biochemistry. Following PPIX's first total synthesis in the late 1920s, synthesis of the core and modification of the substituents of this natural porphyrin have been of keen interest. The propionic acid moieties, vinyl groups and meso‐positions of PPIX have been subjected to transformation to introduce functionalities such as solubilizing groups, drugs, targeting and interacting moieties, or to incorporate the porphyrin into polymers and for immobilization on surfaces.

PPIX and especially its metal complexes serve as functional entities in a plethora of chemical biology applications, some of which have been described in this review. PPIX and its Zn(II) complex act as fluorescent probes in DNA‐G‐quadruplex‐based biosensors. Those have been applied for the detection of nucleic acids, proteins, metal ions, small molecules such as toxins and the monitoring of biochemical processes. The versatility of their applicability implies that in future porphyrin‐G‐quadruplexes will find entry in smart sensing devices such as biochips.

PPIX's natural significance as the macrocycle of heme allows it to easily interact with certain biomolecules as well as to enter into and interfere with biological compartments. Incorporation of non‐natural metalloporphyrins into hemoproteins renders them as catalysts for redox and electrochemical reactions as well as carbon‐carbon and carbon‐heteroatom bond formation reactions in vitro. The catalytic activities of these biohybrids can be tuned by the insertion of different metals and modification of the coordination sphere through directed evolution of the protein scaffold. In addition, it is conceivable that synthetic changes to the porphyrin skeleton can be used to modulate binding and orientation of the cofactor in the protein sphere and enhance interactions with substrate molecules, an approach less explored thus far. This might lead to the development of catalysts that show higher catalytic activities and expanded substrate scopes compared to native heme enzymes.

PPIX metal complexes have also been tested as potential antibiotics by exploiting the natural demand of pathogenic microbes for heme‐iron. Tricking these organisms' heme uptake systems into binding and transporting heme analogues can cause fatality either by reduced heme uptake or by the inherent adverse properties of the metalloporphyrins. Such porphyrins could also be exploited as delivery systems for covalently attached drugs to be acquired by bacteria.

PPIX has found many more applications in various fields such as PDT, imaging, sensing, light harvesting, biomodulation, catalysis and supramolecular chemistry which could not be discussed in detail herein. Furthermore, there is a plethora of uses of native heme and other closely PPIX‐related porphyrins such as mesoporphyrin that we could not cover in this review. One classic and noteworthy example in this respect is the development of catalytic antibodies and DNAzymes against *N*‐methyl‐mesoporphyrin as a transition state analogue for porphyrin metalation und use of these antibodies to catalyze metal insertions into mesoporphyrin.^97^, ^147^

Clearly, PPIX is a molecular scaffold with significant potential in biomedical and materials sciences. Synthetic modifications can improve the performance in existing systems and will further transform uses of the red pigment of life in the development of new applications.
